# Diversity, Local Utilization, and Ethnolinguistic Perspectives on Verbenaceae J.St.-Hil. in Community Landscapes of Buri Ram Province, Eastern Thailand

**DOI:** 10.3390/life16101745

**Published:** 2026-10-17

**Authors:** Surapon Saensouk, Piyaporn Saensouk, Thawatphong Boonma, Suriya Phimpha, Wei Shen, Pathomthat Srisuk, Auemporn Junsongduang

**Affiliations:** 1 Diversity of Family Zingiberaceae and Vascular Plant for Its Applications Research Unit, Walai Rukhavej Botanical Research Institute, Mahasarakham University, Maha Sarakham 44150, Thailand; boonma.thawat@gmail.com; 2 Diversity of Family Zingiberaceae and Vascular Plant for Its Applications Research Unit, Department of Biology, Faculty of Science, Mahasarakham University, Maha Sarakham 44150, Thailand; 3 Faculty of Humanities and Social Sciences, Mahasarakham University, Maha Sarakham 44150, Thailand; suriya.pi@msu.ac.th; 4 Agriculture and Food Engineering College, Baise University, Baise 533001, China; bsxyshenwei@bsuc.cn; 5 Department of Pharmaceutical Technology, Faculty of Pharmaceutical Sciences, Khon Kaen University, Khon Kaen 40002, Thailand; spatho@kku.ac.th; 6 Program of Biology, Department of Science and Technology, Faculty of Liberal Arts and Science, Roi Et Rajabhat University, Ko Kaeo, Selaphum, Roi Et 45120, Thailand; a.junsongduang@reru.ac.th

**Keywords:** community landscapes, ethnobotany, medicinal plants, eastern Thailand, ornamental, species diversity, traditional knowledge, Verbenaceae

## Abstract

Verbenaceae are prominent components of cultivated and human-influenced landscapes, yet their taxonomic and horticultural diversity and relationships with local plant knowledge remain poorly documented in Buri Ram Province, Thailand. This study provides an integrated province-wide assessment of Verbenaceae across all 23 districts, combining voucher-based taxonomy and ecological occurrence with community recognition, utilization, cultural importance, and vernacular nomenclature. Field surveys and interviews with 460 informants documented 16 taxa in eight genera, including species, an interspecific hybrid, named cultivars, and horticultural variants. Regarding their biogeographical status in Thailand, 15 recorded taxa were classified as introduced, whereas *Phyla nodiflora* was the only native taxon. Ornamental use dominated, accounting for 1370 of 1405 use reports, whereas medicinal uses were recorded only for *P. nodiflora* and *Stachytarpheta jamaicensis*. Twenty-two unique vernacular names, including Thai and Thai-Khmer forms, frequently reflected morphological or sensory characteristics and horticultural terminology. Recognition, reported utilization, and vernacular differentiation did not necessarily correspond with taxonomic or horticultural diversity. By integrating voucher-based botanical documentation with horticultural diversity and community-derived knowledge, this study complements conventional floristic approaches and provides baseline evidence for understanding plant diversity and human–plant relationships in Southeast Asian community landscapes.

## 1. Introduction

The family Verbenaceae J.St.-Hil. comprises herbs, shrubs, and small trees of considerable ornamental, medicinal, ecological, and economic importance [1,2,3,4,5,6]. Members are generally characterized by opposite leaves, racemose, spicate, or capitate inflorescences, predominantly bisexual flowers with 4–5-merous corollas, and dry or fleshy fruits [2,5,6,7,8,9,10,11,12,13]. Globally, the family currently includes 33 accepted genera and approximately 900 accepted species [3,14]. The family is predominantly distributed in tropical and subtropical regions, with its greatest diversity in the Americas, particularly Central and South America, although some members also occur in temperate regions of both hemispheres [2,3]. Many members have been introduced beyond their native ranges through horticulture and other human activities, and some may escape cultivation and become established in disturbed and human-managed environments [14,15,16].

In Thailand, six species of Verbenaceae have been formally documented in the Flora of Thailand [7]. Current distributional information recognizes *Phyla nodiflora* (L.) Greene and *Verbena officinalis* L. as native to Thailand [14], whereas several other members are introduced, cultivated, or naturalized. However, conventional floristic treatments do not necessarily encompass the full diversity encountered in contemporary community landscapes, particularly horticultural cultivars, hybrids, and ornamental variants. Such environments may therefore contain taxonomic and horticultural diversity that complements conventional records of the wild flora.

Studies from other parts of Southeast Asia have also demonstrated the importance of home gardens as components of plant diversity in human-managed landscapes. In Indonesia, surveys of urban home gardens in Bandung documented a substantial representation of ornamental plants, while research near Kerinci Seblat National Park recorded diverse plant assemblages associated with food, medicinal, and ornamental uses. These findings highlight the importance of considering cultivated plants and locally reported uses when documenting plant diversity beyond natural habitats [17,18].

Verbenaceae are also closely associated with human use and local plant knowledge. Some taxa are widely cultivated as ornamentals, whereas others are used in traditional medicine and other local practices [1,4]. Vernacular plant names may reflect morphology, color, habit, use, origin, or other culturally salient attributes, thereby providing insights into how people perceive and classify plant diversity [19,20,21]. Importantly, vernacular classification does not necessarily correspond directly with scientific taxonomy: several scientifically distinguishable entities may be grouped under a single folk taxon, whereas a single scientific species may be subdivided into multiple folk taxa based on locally meaningful characteristics [19]. Such relationships provide an opportunity to explore how taxonomic and horticultural diversity is recognized within local systems of plant knowledge.

This perspective is particularly relevant in Buri Ram, where communities occur within a culturally and linguistically diverse setting. Previous research on Passifloraceae in Non Din Daeng District demonstrated that vernacular plant names can reflect local classification and cultural perceptions and may incorporate different linguistic influences [22]. That study applied an ethnolinguistic framework in which plant names were interpreted according to proper, metonymical, and metaphorical meanings [21,22]. This provides a useful basis for exploring how vernacular naming relates to plant diversity in other families occurring in Buri Ram. Rather than assuming rigid correspondence between plant knowledge and discrete ethnolinguistic groups, documenting names as they are currently used within communities may better reflect contemporary patterns of cultural and linguistic interaction.

Despite the ecological, horticultural, and cultural relevance of Verbenaceae, information from Buri Ram remains limited. *Lantana camara* L. and *L. trifolia* L. have been reported from the province in the Flora of Thailand [7], while *Stachytarpheta jamaicensis* (L.) Vahl was subsequently documented by Saensouk et al. [23]. However, cultivated taxa, horticultural variants, naturalized elements, community recognition, local utilization, cultural importance, and vernacular nomenclature remain poorly documented at the provincial level. Community landscapes, including home gardens, village surroundings, roadsides, agricultural areas, and associated disturbed habitats, therefore provide an important setting for documenting an often overlooked component of regional plant diversity and its relationships with local communities.

Accordingly, this study aimed to investigate the diversity, community recognition, and local utilization of Verbenaceae in community landscapes of Buri Ram, Eastern Thailand, while incorporating ecological and ethnolinguistic perspectives. Specifically, we aimed to (1) document the taxonomic and horticultural diversity of Verbenaceae in community landscapes; (2) assess community recognition and local utilization of the recorded taxa; (3) evaluate their cultural importance, particularly in ornamental and traditional medicinal uses; and (4) document vernacular and alternative local names and examine their relationships with taxonomic and horticultural diversity. This study provides voucher-based and community-derived baseline information relevant to floristics, horticulture, ethnobotany, and ethnolinguistics and contributes to a broader understanding of the relationships between plant diversity and local knowledge in human-influenced landscapes. However, the study was restricted to community and other human-influenced landscapes and did not include systematic surveys of natural forests or protected areas. Furthermore, the purposive selection of informants limits the generalizability of the ethnobotanical findings to the wider provincial population.

## 2. Materials and Methods

### 2.1. Study Area and Field Surveys

**Figure 1:**
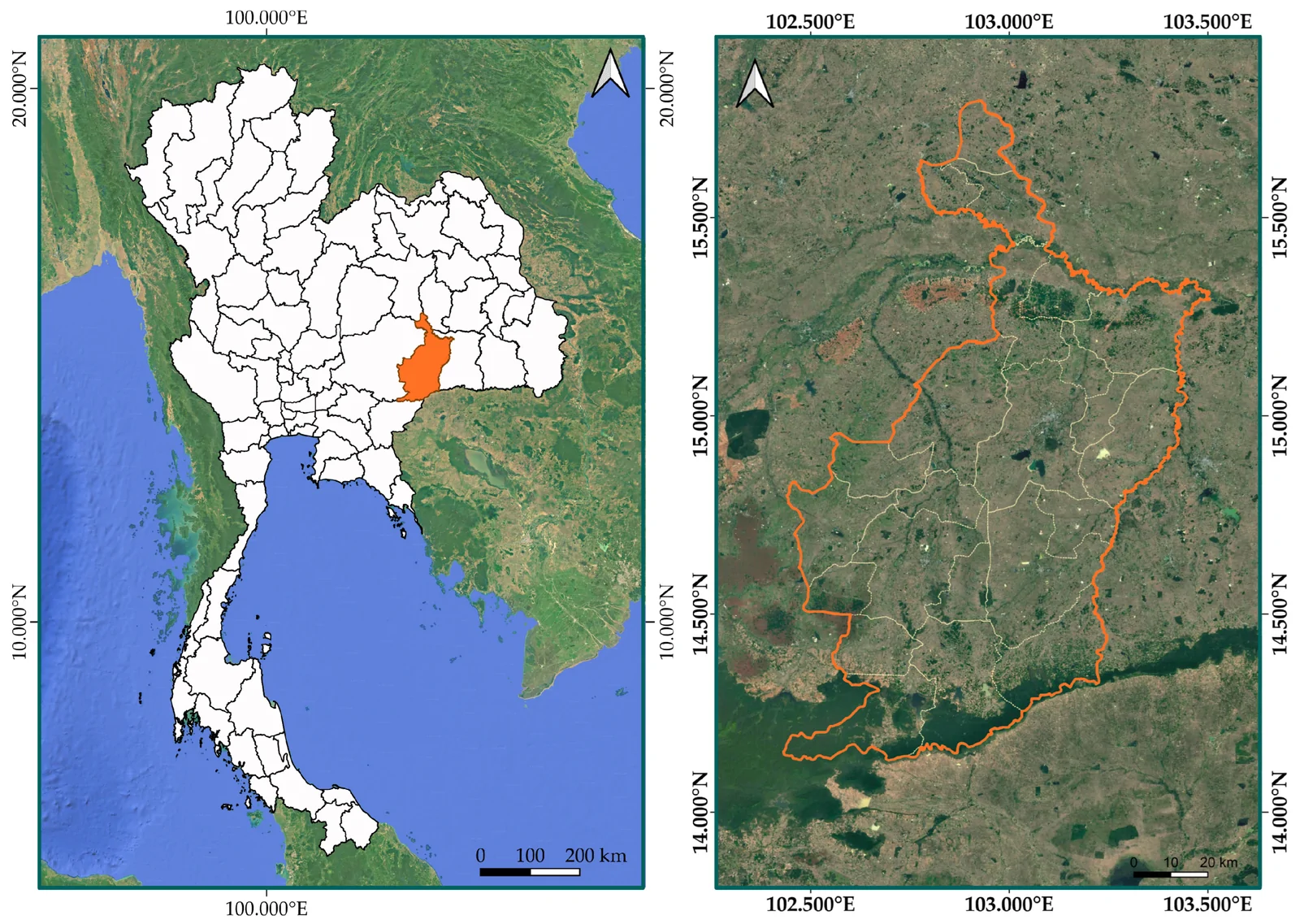
Location of the study area in Buri Ram, Eastern Thailand. The (**left**) panel shows the location of Buri Ram within Thailand, while the (**right**) panel shows the provincial boundary and administrative districts. Field surveys of Verbenaceae in community landscapes were conducted across all 23 districts. The distribution map was prepared using QGIS version 3.34 with the WGS 84 geographic coordinate reference system (EPSG:4326) by Kamonwan Koompoot.

Field surveys were conducted throughout all 23 districts of Buri Ram Province, Eastern Floristic Region, Thailand (Figure 1), from February to September 2026. The province extends approximately from 14°15′ to 15°45′ N and from 102°30′ to 103°45′ E, covering an area of approximately 10,394 km^2^. Its climate is classified as tropical savanna (Aw) under the Köppen–Geiger climate classification, characterized by distinct wet and dry seasons [22,24]. The surveys focused on community landscapes and other human-influenced environments, including home gardens, village surroundings, roadsides, agricultural areas, wetlands, and associated disturbed habitats. Protected areas and natural forests were excluded from the scope of the study. According to official provincial statistics, the economy of Buri Ram is characterized by service, agricultural, and industrial activities, which accounted for approximately 56%, 25%, and 19% of the gross provincial product (GPP), respectively, in 2024 [25]. Agriculture includes the cultivation of jasmine rice, cassava, sugarcane, and rubber, while service-related activities contribute substantially to the provincial economy. These socioeconomic characteristics provide general context for the human-influenced landscapes investigated in this study [25].

Verbenaceae encountered during the surveys were documented through direct field observation, photographed, and collected for taxonomic examination and voucher preparation. Ecological occurrence was recorded according to the conditions under which each taxon was observed, including cultivated, naturalized, and spontaneous occurrences. These occurrences were not treated as mutually exclusive; when the same taxon was observed under more than one condition, all applicable occurrences were recorded. The status of each taxon in Thailand as native or introduced was treated separately from its observed ecological occurrence.

### 2.2. Taxonomic and Horticultural Identification and Voucher Specimens

Taxonomic identification was based primarily on vegetative and reproductive morphological characters. Specimens were identified and compared using the relevant taxonomic literature, identification keys, regional floristic treatments, and taxonomic revisions of Verbenaceae [2,7,8,9,10,11,12,13,14]. Accepted scientific names, nomenclatural status, and geographical distributions were subsequently verified using Plants of the World Online (POWO) [14].

Because the diversity encountered in community landscapes included species, hybrids, cultivars, and recognizable horticultural variants, the collective term “taxa” was used where appropriate. Formally named cultivars were recorded under their cultivar names when their identity could be established. The identities of the cultivated *Lantana camara* cultivars ‘Mutabilis’, ‘Nivea’, and ‘Sundancer’ were additionally checked using the Flora & Fauna Web of the National Parks Board, Singapore (NParks). Recognizable horticultural variants without formal cultivar names, including the pink-, purple-, and red-flowered variants of *Glandularia tenera*, were documented descriptively and were not treated as separate species or formally named cultivars.

Representative specimens of all recorded taxa were prepared as herbarium vouchers and deposited in the Vascular Plant Herbarium, Mahasarakham University (VMSU). Voucher numbers are provided in Table 1.

### 2.3. Community Recognition and Ethnobotanical Data Collection

Ethnobotanical data were collected from 460 local informants, with 20 informants purposively selected from each of the 23 districts of Buri Ram. Informants were selected from residents living in or near sites where Verbenaceae were recorded and who were familiar with plants occurring in their local surroundings. All informants were presented with the Verbenaceae taxa documented during the province-wide field survey. Living plants, where available, or photographs taken during the field surveys were used to facilitate recognition and ensure that questions referred to the intended taxon. For each taxon, informants were first asked whether they recognized the plant. When a plant was recognized, subsequent questions documented its vernacular name, alternative local or dialect names, and whether it was locally used. When a use was reported, follow-up questions recorded the purpose of use, plant part used, and, where applicable, the method of preparation or application. No further questions concerning nomenclature or utilization were asked when an informant did not recognize the plant. Interviews focused exclusively on plant-related knowledge. No demographic information, including age, sex, or ethnicity, was collected because the study aimed to document community recognition, plant utilization, and local nomenclature rather than compare knowledge among demographic or ethnolinguistic groups. Vernacular and dialect names were therefore recorded as reported by informants without a priori attribution to particular ethnic groups. Before each interview, participants were informed of the purpose of the study, the voluntary nature of participation, and their right to withdraw. Verbal informed consent was obtained from all participants. The interview protocol was approved by the Human Ethics Committee of Mahasarakham University. The study was conducted in accordance with relevant institutional guidelines and the principles of the International Society of Ethnobiology (ISE) Code of Ethics and the Nagoya Protocol on Access and Benefit Sharing. A blank participant information and verbal consent script, including the interview questions and their sequence, is provided in Supplementary File S1.

### 2.4. Ethnolinguistic Documentation and Analysis

Vernacular plant names were documented in the forms reported by informants. During the interviews, informants were asked to pronounce each vernacular plant name syllable by syllable to facilitate accurate phonetic transcription. The pronunciations were transcribed using the International Phonetic Alphabet (IPA) during the interviews, based on the syllabic pronunciations provided by the informants. Particular attention was given to alternative local or dialect names, the use of the same name for multiple botanical entities, and cases in which different names were applied to species, cultivars, or horticultural variants. Where sufficient linguistic and contextual information was available, vernacular names were interpreted following ethnolinguistic approaches previously applied to Thai plant nomenclature [20,21,22]. Names were examined in terms of proper, metonymical, and metaphorical meanings and, where applicable, in relation to characteristics such as plant morphology, color, habit, use, origin, or other culturally meaningful associations. Linguistic origins or cultural interpretations were not assigned when supporting information was insufficient. Etymological explanations of vernacular names were recorded according to informants’ interpretations. Where the historical or linguistic origin of a term could not be independently verified, it was treated as a local interpretation rather than as a definitive etymological derivation.

### 2.5. Data Analysis

Taxonomic and horticultural diversity was summarized in terms of the numbers of genera, species, hybrids, cultivars, and recognizable horticultural variants recorded during the province-wide surveys. The status of each taxon in Thailand was determined using the relevant floristic literature and current distributional information from POWO [7,14]. Ecological occurrence was summarized directly from field observations, allowing cultivated, naturalized, and spontaneous occurrences to overlap when more than one condition was documented for the same taxon.

#### 2.5.1. Community Recognition Rate

Community recognition of each taxon was evaluated using the proportion of informants who recognized it. Because all 460 informants were presented with the same set of Verbenaceae taxa recorded during the province-wide survey, the Recognition Rate (RR) was calculated as:

(1) $${RR}_{s}=\;\frac{R_{s}}{N}\times 100$$

where *RR_s_* is the Recognition Rate (%) of taxon *s*, *R_s_* is the number of informants who recognized taxon *s*, and *N* is the total number of informants interviewed (*N* = 460). Recognition was analyzed separately from utilization; therefore, recognition of a plant did not itself constitute a use report.

#### 2.5.2. Cultural Importance Index

The Cultural Importance Index (*CI*) was used to evaluate the relative cultural importance of each taxon among the sampled informants based on the frequency and diversity of reported uses. Two use categories emerged from the interview data: ornamental use and traditional medicinal use. Ornamental use was recorded only when an informant reported that a plant was cultivated, maintained, or otherwise used for ornamental purposes; recognition of a plant as ornamental without reported use was not counted as a use report.

For each taxon, one use report per informant per use category was counted, irrespective of the number of specific applications reported within that category. Category-specific cultural importance was calculated as:

(2) $${CI}_{u,s}=\;\frac{{UR}_{u,s}}{N}$$

where *CI_u,s_* is the cultural importance of taxon *s* within use category *u*. *UR_u,s_* is the number of informants reporting taxon *s* within that use category, and *N* is the total number of informants interviewed (*N* = 460).

The overall Cultural Importance Index for each taxon was calculated as the sum of the category-specific values:

(3) $${CI}_{s}=\;\sum_{u=1}^{2}\frac{{UR}_{u,s}}{N}$$

where the two use categories were ornamental and traditional medicinal use. Category-specific *CI_u,s_*values were retained to distinguish the relative contribution of each use category to the overall *CI_s_* of each taxon.

#### 2.5.3. Fidelity Level

The Fidelity Level (FL) was calculated only for taxa for which traditional medicinal uses were reported. FL was used to determine the degree to which medicinal-use informants associated a particular taxon with a specific medicinal purpose or ailment. It was calculated as:

(4) $$FL(\%)=\;\frac{I_{p}}{I_{u}}\times 100$$

where *I_p_* is the number of informants reporting the use of a particular taxon for a specific medicinal purpose or ailment, and *I_u_* is the total number of informants reporting any medicinal use of that taxon.

An informant reporting several preparation or application methods for the same medicinal purpose was counted once for that purpose. When an informant reported the same taxon for more than one medicinal purpose, the informant could contribute to each relevant purpose but was counted only once in *I_u_*. FL values were interpreted together with the corresponding numbers of informants to avoid overinterpretation of high percentages based on small numbers of reports.

### 2.6. Research Timeline and Data Processing

Field surveys and ethnobotanical interviews were conducted concurrently across all 23 districts of Buri Ram Province from February to September 2026. Taxonomic identification, specimen processing, and voucher deposition at the Vascular Plant Herbarium, Mahasarakham University (VMSU), were undertaken progressively throughout the fieldwork period. Voucher specimens were deposited under accession numbers VMSU38200004–VMSU38200019. Vernacular names were transcribed using the International Phonetic Alphabet (IPA) during interviews, while data entry, verification, and quantitative analyses were performed alongside ongoing fieldwork as records became available. Following the completion of fieldwork in September 2026, the accumulated records and analytical results were reviewed and consolidated, and the manuscript was prepared during September–October 2026.

## 3. Results

### 3.1. Diversity of Verbenaceae in Community Landscapes of Buri Ram

**Figure 2:**
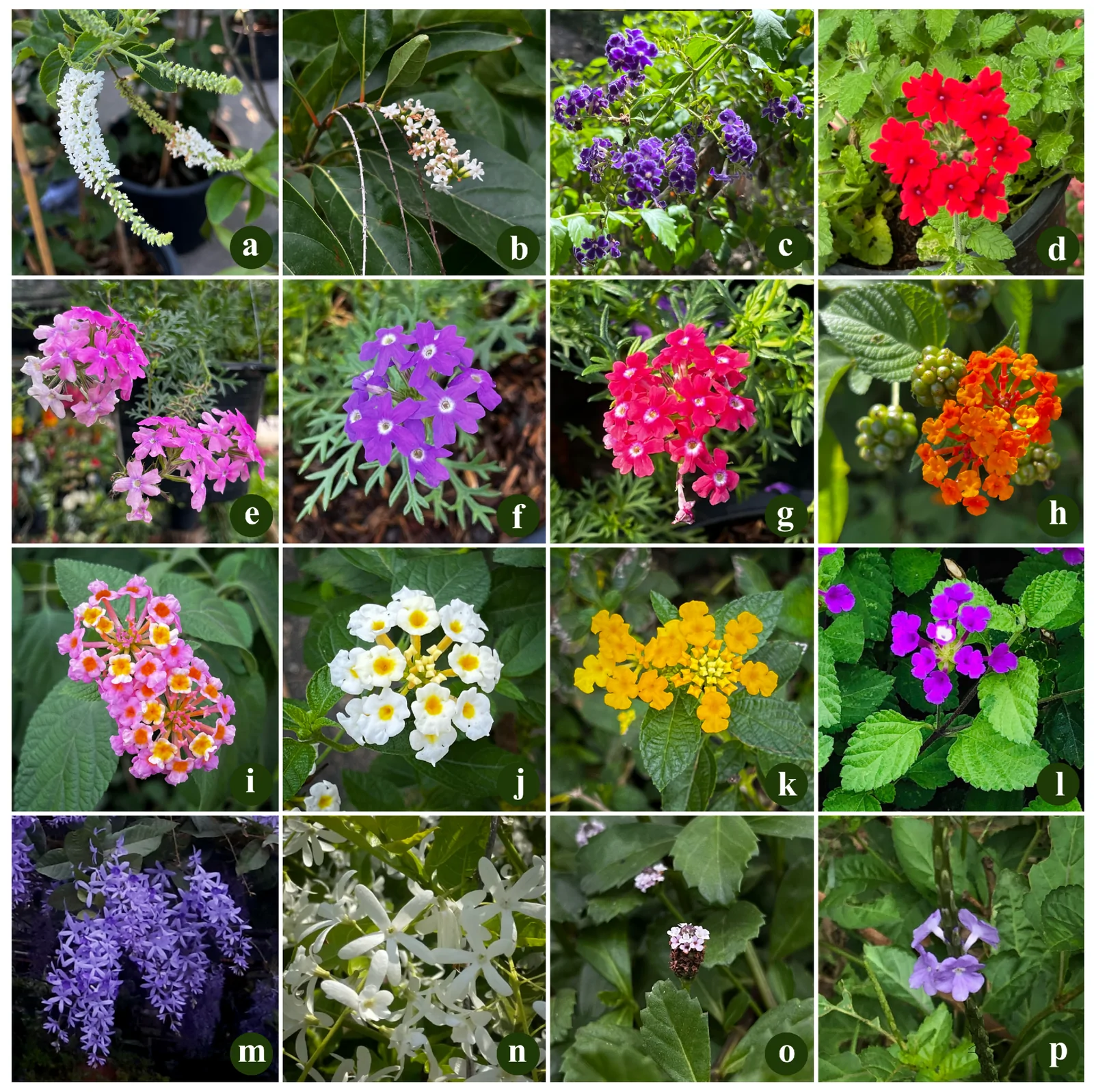
Verbenaceae recorded in community landscapes of Buri Ram Province, Eastern Thailand: (**a**) *Aloysia virgata*; (**b**) *Citharexylum spinosum*; (**c**) *Duranta erecta* ‘Dark Purple’; (**d**) *Glandularia × hybrida*; (**e**) *Glandularia tenera* (pink-flowered variant); (**f**) *G. tenera* (purple-flowered variant); (**g**) *G. tenera* (red-flowered variant); (**h**) *Lantana camara*; (**i**) *L. camara* ‘Mutabilis’; (**j**) *L. camara* ‘Nivea’; (**k**) *L. camara* ‘Sundancer’; (**l**) *L. montevidensis*; (**m**) *Petrea volubilis*; (**n**) *P. volubilis* ‘Albiflora’; (**o**) *Phyla nodiflora*; (**p**) *Stachytarpheta jamaicensis*. Photographs by Thawatphong Boonma.

**Figure 3:**
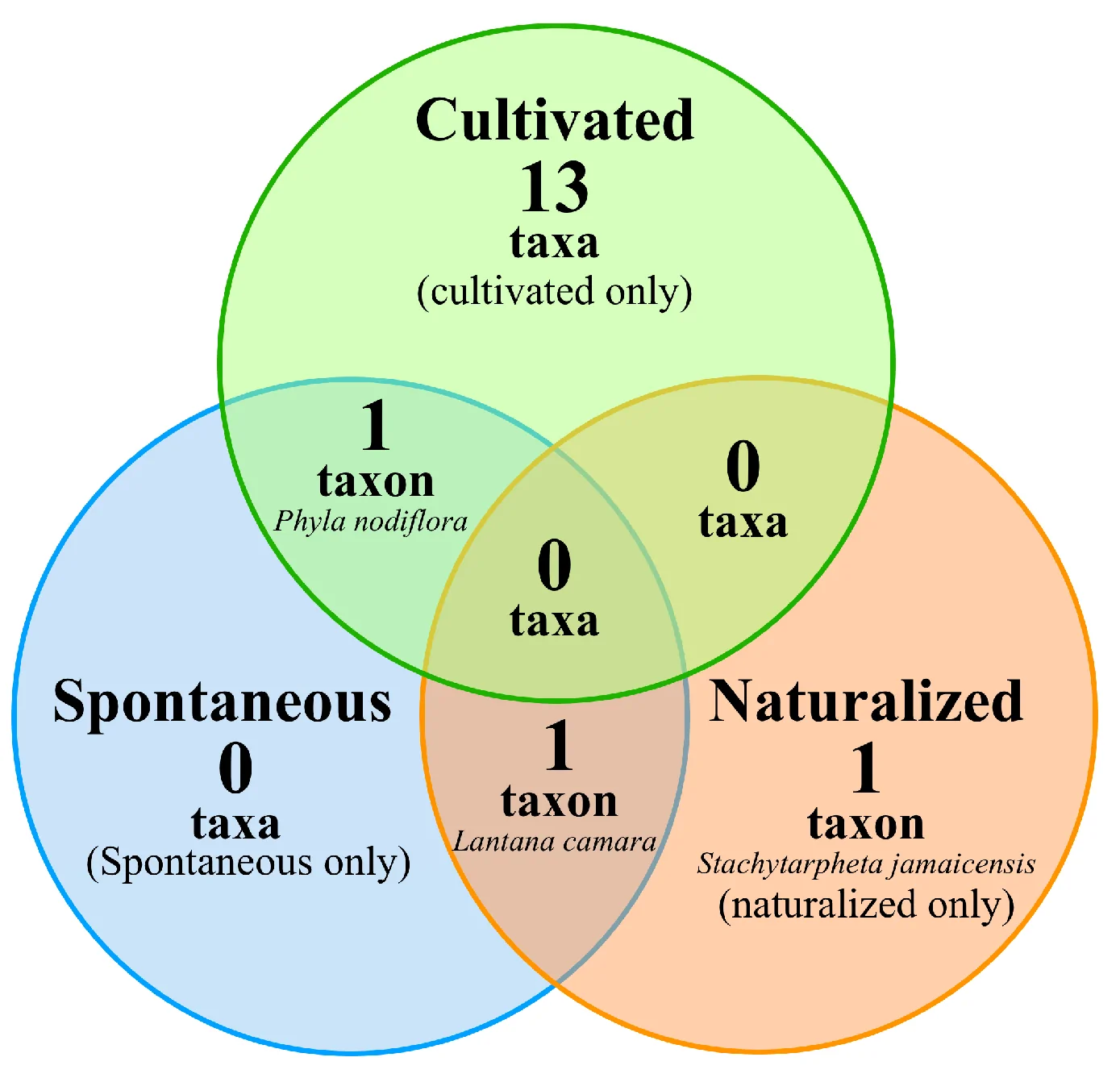
Venn diagram showing the ecological occurrences of the 16 Verbenaceae taxa recorded in community landscapes of Buri Ram. Numbers indicate the number of taxa recorded as cultivated, naturalized, spontaneous, or under overlapping occurrence conditions.

A total of 16 taxa representing eight genera of Verbenaceae were documented in community landscapes of Buri Ram, Eastern Thailand (Figure 2 and Figure 3; Table 1).

The recorded diversity comprised species, an interspecific hybrid, formally named cultivars, and recognizable horticultural variants. Of the 16 recorded taxa, 15 were introduced to Thailand, whereas *Phyla nodiflora* (L.) Greene was native. Four taxa were previously documented for Thailand in the Flora of Thailand, namely *Duranta erecta*, *Lantana camara*, *Phyla nodiflora*, and *Stachytarpheta jamaicensis*, whereas the remaining recorded taxa, including horticultural entities, were not included in that treatment.

Eight genera were represented: *Aloysia*, *Citharexylum*, *Duranta*, *Glandularia*, *Lantana*, *Petrea*, *Phyla*, and *Stachytarpheta*. *Lantana* showed the greatest horticultural representation, comprising two species and three named cultivars of *L. camara*. *Glandularia* was represented by the interspecific hybrid *G. × hybrida* and *G. tenera*, with the latter represented by three recognizable flower-color variants (pink, purple, and red). *Petrea* was represented by *P. volubilis* and the cultivar ‘Albiflora’, whereas *Aloysia*, *Citharexylum*, *Duranta*, *Phyla*, and *Stachytarpheta* were each represented by a single recorded taxonomic or horticultural entity.

### 3.2. Ecological Occurrence in Community Landscapes

Ecological occurrence varied among the recorded Verbenaceae, although cultivation was the predominant condition. Fourteen of the 16 recorded taxa were observed under cultivation. Of these, 13 taxa were recorded exclusively as cultivated plants, whereas *Phyla nodiflora* occurred both spontaneously and under cultivation. Among taxa occurring outside cultivation, *Lantana camara* was recorded as both naturalized and spontaneous, while *Stachytarpheta jamaicensis* was recorded as naturalized. The Verbenaceae assemblage in community landscapes of Buri Ram was strongly dominated by cultivated taxa, with only three taxa showing spontaneous and/or naturalized occurrences (Table 1).

### 3.3. Community Recognition of Verbenaceae

Community recognition varied considerably among the 16 Verbenaceae taxa recorded in Buri Ram. *Petrea volubilis* showed the highest recognition and was recognized by all 460 informants (RR = 100%). High recognition was also recorded for *P. volubilis* ‘Albiflora’ (*n* = 430; RR = 93.5%), *Lantana camara* ‘Mutabilis’ (*n* = 422; RR = 91.7%), *L. camara* (*n* = 408; RR = 88.7%), *L. camara* ‘Sundancer’ (*n* = 389; RR = 84.6%), and *Duranta erecta* ‘Dark Purple’ (*n* = 375; RR = 81.5%).

Intermediate levels of recognition were recorded for *L. camara* ‘Nivea’ (RR = 73.3%), *L. montevidensis* (72.4%), and *Stachytarpheta jamaicensis* (68.0%). In contrast, recognition was lower for *Phyla nodiflora* (39.3%), *Aloysia virgata* (23.7%), and *Citharexylum spinosum* (21.3%). The lowest recognition rates were recorded among *Glandularia* taxa, ranging from 14.3% for the pink-flowered variant of *G. tenera* to 17.0% for the purple-flowered variant. These results demonstrate substantial differences in the extent to which the recorded taxonomic and horticultural diversity was recognized by local communities (Table 2).

### 3.4. Local Utilization and Cultural Importance

**Figure 4:**
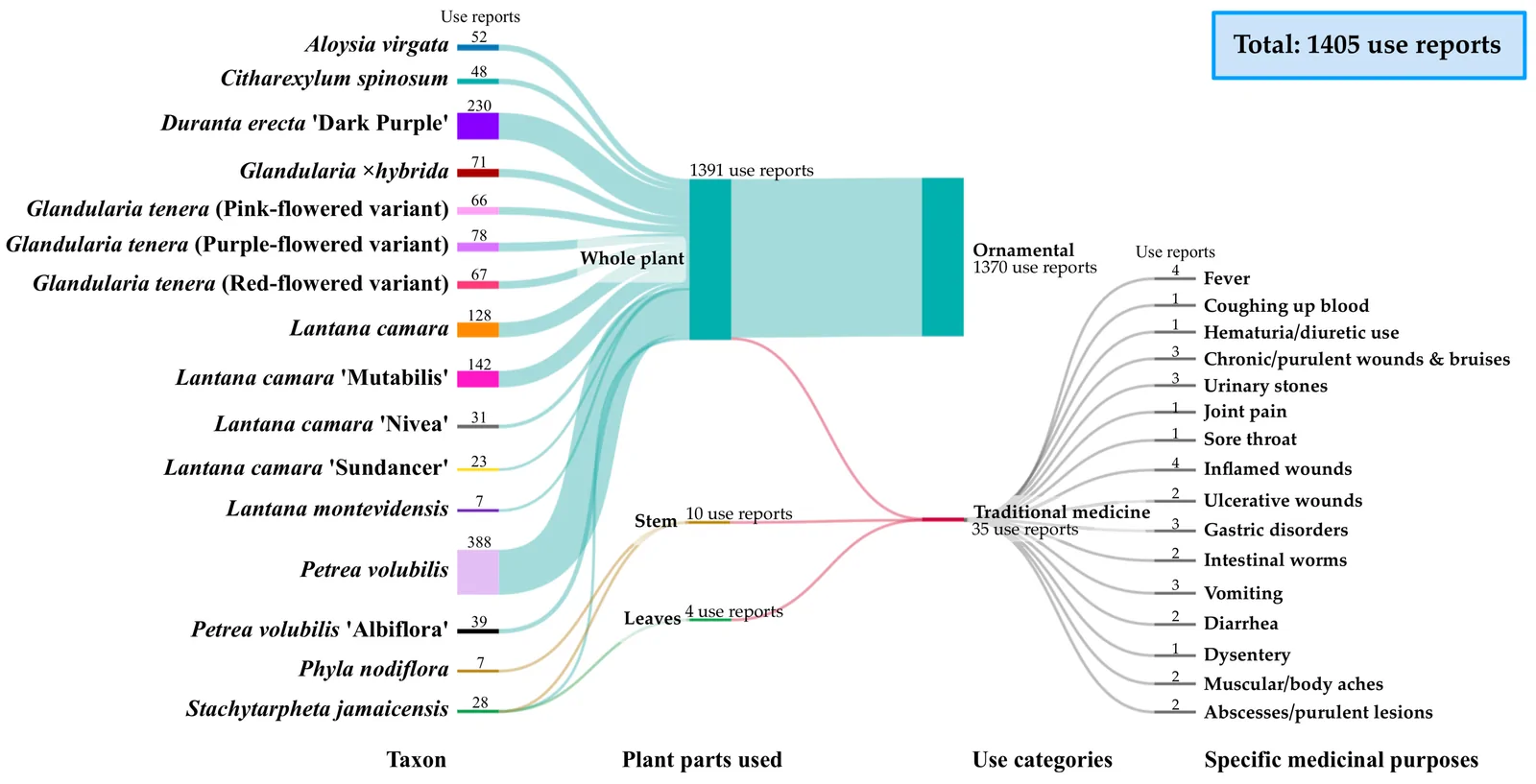
Sankey diagram illustrating local utilization patterns of Verbenaceae in community landscapes of Buri Ram, Eastern Thailand. Flows connect individual taxa with plant parts used, use categories, and specific medicinal purposes, with flow width proportional to the number of use reports (UR) recorded from 460 informants. A total of 1405 use reports were documented, comprising 1370 ornamental and 35 traditional medicinal use reports.

A total of 1405 use reports (UR) were documented for the 16 Verbenaceae taxa recorded in community landscapes of Buri Ram. Local utilization was represented by two use categories: ornamental and traditional medicinal uses. Ornamental use predominated, accounting for 1370 UR across 14 taxa, whereas traditional medicinal use accounted for 35 UR and involved only *Phyla nodiflora* and *Stachytarpheta jamaicensis* (Figure 4).

The Cultural Importance Index (CI) varied considerably among the recorded taxa (Table 2). *Petrea volubilis* had the highest number of ornamental use reports (388 UR) and the highest CI value (0.843), followed by *Duranta erecta* ‘Dark Purple’ (230 UR; CI = 0.500), *Lantana camara* ‘Mutabilis’ (142 UR; CI = 0.309), and *L. camara* (128 UR; CI = 0.278). Lower ornamental CI values were recorded for *L. montevidensis* (7 UR; CI = 0.015), *L. camara* ‘Sundancer’ (23 UR; CI = 0.050), *L. camara* ‘Nivea’ (31 UR; CI = 0.067), and *Petrea volubilis* ‘Albiflora’ (39 UR; CI = 0.085).

Traditional medicinal use was much less frequently reported. *Stachytarpheta jamaicensis* accounted for 28 medicinal UR (CI = 0.061), whereas *Phyla nodiflora* accounted for seven medicinal UR (CI = 0.015). No ornamental use reports were recorded for these two taxa in the interview dataset.

The relationship among taxa, plant parts used, use categories, and reported medicinal purposes is summarized in the Sankey diagram (Figure 4). Of the 1405 UR, the whole plant accounted for 1391 reports, reflecting its predominant use for ornamental purposes, while stems and leaves accounted for 10 and four reports, respectively. All 1370 ornamental UR involved the whole plant. The 35 medicinal UR comprised 21 reports involving the whole plant, 10 involving stems, and four involving leaves. Detailed medicinal purposes, preparation and application methods, and Fidelity Level (FL) values are presented separately in Table 3.

Community recognition did not necessarily correspond with the frequency of reported utilization. For example, *P. volubilis* was recognized by all 460 informants and was also widely reported for ornamental use (388 UR), whereas *P. volubilis* ‘Albiflora’ was recognized by 430 informants but accounted for only 39 ornamental UR. Similarly, *L. montevidensis* was recognized by 333 informants but accounted for only seven ornamental UR. These patterns indicate variation between recognition and reported utilization among the recorded taxa.

### 3.5. Traditional Medicinal Uses and Fidelity Level

Traditional medicinal uses were documented for two Verbenaceae species, *Phyla nodiflora* and *Stachytarpheta jamaicensis*, accounting for 35 use reports (UR) in total (Figure 4; Table 3). *Stachytarpheta jamaicensis* accounted for 28 UR, whereas *P. nodiflora* accounted for seven UR. Medicinal uses involved the whole plant, stems, or leaves and encompassed a range of reported medicinal purposes.

All seven medicinal use reports for *P. nodiflora* involved the stem. The most frequently reported use was for chronic or purulent wounds and bruises (3 UR; FL = 42.9%), followed by fever (2 UR; FL = 28.6%). Coughing up blood and hematuria associated with promotion of urination were each represented by one report (FL = 14.3% each). For internal uses, dried stems were boiled in water, and the resulting decoction was taken orally, whereas treatment of wounds and bruises involved applying the boiled plant material externally as a poultice. All seven informants reporting medicinal use of *P. nodiflora* also stated that oral use should be avoided during pregnancy; this was treated as a reported local precaution and was not counted as an additional use report.

*Stachytarpheta jamaicensis* showed a broader range of reported medicinal uses, with 28 UR distributed among 13 medicinal purposes. The whole plant accounted for 21 UR, stems for three UR, and leaves for four UR. Among whole plant uses, inflamed wounds had the highest FL (4 UR; FL = 14.3%), followed by urinary stones, gastric disorders, and vomiting (3 UR each; FL = 10.7% each). Fever, ulcerative wounds, and intestinal worms were each represented by two reports (FL = 7.1% each), while joint pain and sore throat were each represented by one report (FL = 3.6% each). Stem uses comprised diarrhea (2 UR; FL = 7.1%) and dysentery (1 UR; FL = 3.6%), whereas leaves were reported for muscular or body aches and abscesses or purulent lesions (2 UR each; FL = 7.1% each).

Preparation and application methods varied according to medicinal purpose (Table 3). Oral preparations of *S. jamaicensis* principally involved decoctions or infusions, whereas external applications involved pounding fresh plant material or rubbing or massaging leaves onto the affected area. *Stachytarpheta jamaicensis* exhibited a broader diversity of reported medicinal purposes, whereas medicinal use of *P. nodiflora* was more concentrated, with the highest FL associated with the external treatment of chronic or purulent wounds and bruises.

### 3.6. Vernacular Names and Ethnolinguistic Characteristics

A total of 22 unique vernacular names were recorded for the Verbenaceae taxa investigated in Buri Ram Province (Table 4). Of these, 16 names (72.7%) were reported by informants as names conventionally used within their families and local communities, whereas six (27.3%) were reported as names obtained from plant shops or vendors. The latter comprised Rachawadee, Bu-nga Sahree, and the horticultural name Verbena, including the color-modified forms Verbena Chomphoo, Verbena Muang, and Verbena Daeng. Linguistically, 17 names (77.3%) were Thai, whereas five (22.7%) were identified by informants as being of Thai-Khmer origin. Linguistic attribution refers here to the language or reported linguistic origin of individual plant names rather than to the ethnicity of the informants.

The vernacular names reflected several mechanisms of plant recognition and naming, including direct reference to observable characteristics such as color, fragrance, growth habit, and floral or vegetative morphology; metaphorical comparison with animals or familiar objects; and the adoption or modification of established horticultural names. Some names encoded more than one characteristic. For example, Tian Hom combined the candle-related association represented by Tian with Hom, referring to floral fragrance, whereas Ton Ngu Kiaw combined resemblance to a snake with its green coloration. Similar naming concepts were also expressed across Thai and Thai-Khmer forms, particularly in names referring to squirrel-tail-like inflorescences and candle-related imagery. Detailed semantic interpretations and IPA transcriptions of individual names are presented in Table 4.

Morphological resemblance was particularly evident in the vernacular names of *Aloysia virgata*. The Thai name Hang Kra Rok combines Hang (“tail”) and Kra Rok (“squirrel”), referring to the elongated inflorescences that resemble a squirrel’s tail. The same morphological concept occurred in two names identified by informants as Thai-Khmer, Paga Gatui Gone Groke and Paga Gatui Ganghand. According to informants, Paga means “flower” and Gatui means “tail”, while Gone Groke and Ganghand both refer to a squirrel. Thus, although expressed through different linguistic forms, these names share the concept of a “squirrel-tail flower”. The alternative name Rachawadee represents a different naming pattern. Informants associated this name with a royal connotation and noted that the same name is also applied to *Buddleja paniculata* Wall., an ornamental species with superficially similar inflorescences. Its application to *A. virgata* was therefore locally interpreted as a possible transfer of an established plant name based on perceived similarity.

For Citharexylum spinosum, the name Bu-nga Sahree was explained by informants through the components Bu-nga, meaning “flower”, and Sahree, referring to a sari. The long, pendulous white inflorescences were perceived as resembling the graceful hanging portion of a sari, representing a metaphorical interpretation based on the overall appearance of the inflorescence.

Several vernacular names of *Duranta erecta* ‘Dark Purple’ were structured around the concept of a candle. Tian Yot combines Tian (“candle”) and Yot (“drop”), referring to the rounded orange fruits hanging from the branches that were perceived as resembling drops of candle wax. Tian Hom combines Tian with Hom (“fragrant”), incorporating floral fragrance into the candle-related concept, whereas Dok Tian combines Dok (“flower”) and Tian (“candle”). Corresponding concepts were also expressed in names identified by informants as Thai-Khmer language. Ten Gra Aob was interpreted as “fragrant candle”, whereas Pa Ga Ten was interpreted as “candle flower”. These names demonstrate that morphological resemblance and sensory characteristics can occur together within local naming systems.

Horticultural terminology was particularly evident in *Glandularia*. The name *Verbena* recorded for *Glandularia × hybrida*, was reported as an established name used in the ornamental-plant trade and associated by informants with the former placement of these plants under *Verbena*. For *G. tenera*, the same horticultural name was combined with Thai color terms to distinguish the flower-color variants: Verbena Chomphoo, Verbena Muang, and Verbena Daeng, in which Chomphoo, Muang, and Daeng mean “pink”, “purple”, and “red”, respectively. Flower color therefore functions as a direct distinguishing feature within an otherwise shared horticultural name.

A different pattern was observed in *Lantana*. The single vernacular name Phaga Grong was applied to *L. camara* and all three recorded cultivars, ‘Mutabilis’, ‘Nivea’, and ‘Sundancer’; these repeated applications were counted as one unique vernacular name. Informants interpreted Phaga as “flower” and Grong as referring to the stringing or ornamental arrangement of flowers, reflecting the numerous small flowers densely arranged into rounded inflorescences. In contrast, *L. montevidensis* was distinguished as Phaga Grong Luey, in which Luey refers to its creeping or trailing habit. Thus, local nomenclature distinguished a species with a conspicuously different growth habit but did not distinguish the recorded cultivars of *L. camara*.

Color and inflorescence form were combined in the names of *Petrea volubilis*. Phuang Kram consists of Phuang, referring to a cluster or bunch, and Kram, meaning “indigo”, describing the clustered indigo-colored flowers. The white-flowered cultivar ‘Albiflora’ was called Phuang Kram Khaow, in which the established name Phuang Kram is retained and Khaow (“white”) is added to distinguish its flower color.

Leaf morphology was central to the names recorded for *Phyla nodiflora*. Yah Gled Pla consists of Yah, referring to a weed or herb, Gled (“scale”), and Pla (“fish”), whereas Ton Gled Pla replaces Yah with Ton (“plant”). In both names, Gled Pla (“fish scales”) refers to the perceived resemblance of the leaves to fish scales. The difference between Yah and Ton reflects alternative ways of referring to the plant, while the underlying morphological metaphor remains unchanged.

Parallel naming concepts were also recorded for *Stachytarpheta jamaicensis*. The Thai name Ton Ngu Kiaw consists of Ton (“plant”), Ngu (“snake”), and Kiaw (“green”), referring to the elongated green inflorescence perceived as resembling a green snake. Informants also identified Puaa Keew as a Thai-Khmer name and interpreted its overall meaning as “green snake”. Because the individual lexical components of Puaa Keew were not independently established in the present study, the informants’ interpretation of the complete expression was retained without further subdivision. The two names nevertheless demonstrate a shared conceptual association between inflorescence morphology, color, and a green snake.

Vernacular naming of Verbenaceae in the study area reflected both locally maintained family and community usage and contemporary horticultural exchange. Most names were reported by informants as names conventionally used within their families and communities and commonly reflected recognizable morphological or sensory characteristics of the plants. In contrast, several names associated with cultivated ornamentals, particularly Rachawadee, Bu-nga Sahree, and the Verbena names, were reported as having been obtained from plant shops or vendors, indicating another pathway through which plant names circulate locally. Metaphorical names frequently involved comparisons with animals or familiar objects, whereas metonymical elements commonly referred directly to color, fragrance, or growth habit. Corresponding naming concepts expressed in Thai and Thai-Khmer were particularly evident in the “squirrel-tail” names of *Aloysia virgata*, the candle-related names of *Duranta erecta* ‘Dark Purple’, and the “green snake” names of Stachytarpheta jamaicensis. These patterns show that vernacular plant nomenclature in Buri Ram reflects both locally maintained linguistic knowledge and terminology circulating through the ornamental-plant trade.

Among the 22 unique vernacular names recorded, 16 (72.7%) were reported by informants as names used within their families and local communities, whereas six (27.3%) were reported as names obtained from plant shops or vendors. Linguistically, 17 names (77.3%) were Thai language, while five (22.7%) were identified by informants as Thai-Khmer language. The recorded names frequently encoded observable or perceived plant characteristics, including resemblance to animals or familiar objects, flower color, inflorescence or leaf morphology, fragrance, and growth habit. Several names incorporated more than one characteristic; for example, Tian Hom combined a candle-related metaphor with fragrance, while Ton Ngu Kiaw combined resemblance to a snake with its green coloration. Names obtained through the ornamental-plant trade included Rachawadee, Bu-nga Sahree, and the Verbena names, whereas the remaining names were reported as being conventionally used within families and local communities.

## 4. Discussion

### 4.1. Diversity and Occurrence of Verbenaceae in Community Landscapes

The diversity documented in this study highlights the importance of distinguishing between conventional floristic inventories and inventories of plants occurring in community landscapes. The recorded assemblage included not only species but also an interspecific hybrid, named cultivars, and recognizable horticultural variants. Inclusion of these entities was appropriate to the scope of the present study because they formed part of the Verbenaceae diversity encountered in home gardens, village surroundings, roadsides, and other human-influenced environments. The predominance of introduced taxa also reflects the strong representation of cultivated ornamentals within the surveyed landscapes. Current distributional information recognizes *Phyla nodiflora* as native to Thailand, whereas the other recorded taxa are introduced [14].

Comparable studies of home gardens in other parts of Southeast Asia have highlighted the contribution of human-managed environments to local plant diversity. In Indonesia, studies conducted in Bandung, West Java, and in communities near Kerinci Seblat National Park documented diverse home-garden plant assemblages associated with ornamental and other household uses [17,18]. These findings are consistent with the present study in demonstrating that plants maintained within community landscapes constitute an important component of locally encountered botanical diversity. However, whereas those studies examined home-garden plant assemblages more broadly, the present investigation focused specifically on Verbenaceae across several types of community and human-influenced habitats. The predominance of introduced ornamental taxa in our survey further illustrates the importance of distinguishing cultivated and horticultural diversity from native floristic diversity when documenting plants associated with contemporary communities.

Comparison with existing floristic information requires particular caution. The Flora of Thailand documents six species of Verbenaceae nationally [7], whereas only four of the taxa encountered in the present study *Duranta erecta*, *Lantana camara*, *Phyla nodiflora*, and *Stachytarpheta jamaicensis* are included in that treatment. The additional taxa encountered here should not automatically be regarded as new records for Thailand. Several represent cultivated species, hybrids, cultivars, or horticultural variants, and their absence from a conventional flora may reflect differences in scope and taxonomic treatment rather than previously undocumented occurrence in the country [7,14,26,27,28]. The present findings therefore complement conventional floristic information by documenting taxonomic and horticultural diversity encountered in contemporary community landscapes.

The same distinction is important when considering occurrence records for Buri Ram Province. Previous published information cited in the present study documents *Lantana camara* and *L. trifolia* from the province in the Flora of Thailand [7], while *Stachytarpheta jamaicensis* was subsequently documented from Ban Dan District [23]. The present survey therefore broadens voucher-supported documentation of Verbenaceae encountered in community landscapes across all 23 districts. However, taxa without previous records in the sources examined should be interpreted as not documented in those sources, rather than automatically as first records for Buri Ram. Establishing a formal new provincial record, particularly for long-cultivated ornamental plants, would require a more exhaustive examination of herbarium collections, the historical literature, horticultural records, and other occurrence sources before the absence of a taxon from an existing flora can be interpreted as evidence of a previously undocumented provincial occurrence [29].

The field occurrence categories further distinguish the predominantly cultivated component from taxa also encountered outside exclusively cultivated settings. *Phyla nodiflora* occurred both spontaneously and under cultivation, while *Lantana camara* and *Stachytarpheta jamaicensis* were encountered under naturalized and/or spontaneous conditions. The occurrence of *L. camara* outside cultivation is consistent with its well-documented ecological behavior elsewhere in its introduced range [16]. Beyond this comparison, however, the categories recorded in the present study describe the conditions under which plants were observed during field surveys and should not be interpreted as evidence of temporal processes such as escape from cultivation, establishment, or progression toward naturalization. Demonstrating such processes would require population-level and temporal observations beyond the scope of this study.

The horticultural representation of *Lantana* and *Glandularia* further illustrates the relevance of recognizing entities below or outside the conventional species level when investigating community landscapes. Cultivars, hybrids, and recognizable variants may not represent additional wild species, but they constitute distinguishable components of the plant diversity encountered and recognized in human-managed environments. Their documentation is therefore relevant to a study integrating botanical diversity with community recognition and local nomenclature [30,31].

### 4.2. Community Recognition, Utilization, and Cultural Importance

Community recognition and reported utilization provided complementary rather than interchangeable measures of local relationships with the recorded plants. The results showed that high recognition did not invariably correspond to a similarly high number of use reports. In this study, recognition indicates whether a taxon was known to the sampled informants, whereas use reports and the Cultural Importance Index reflect its reported utilization. Considering these measures separately therefore avoids equating knowledge of a plant with its reported cultural use [32,33].

The strong predominance of ornamental use is consistent with both the composition of the recorded assemblage and the community-landscape setting of the study. Most taxa were introduced plants encountered under cultivation, and ornamental uses accounted for the great majority of recorded use reports. This pattern should be interpreted specifically within the Verbenaceae and landscape types investigated here rather than as evidence that ornamental use predominates over other categories of plant use throughout Buri Ram. Previous ethnobotanical investigations in the province have documented broader relationships between communities and plants, including medicinal and other forms of utilization [22,23]. The present findings instead characterize the role of Verbenaceae within the surveyed community landscapes.

Differences in the Cultural Importance Index (CI) among ornamental taxa indicate variation in the frequency and distribution of reported uses among the sampled informants. These differences did not necessarily correspond to patterns of community recognition or the horticultural diversity documented during field surveys. However, the present study did not quantify local plant abundance, planting frequency, duration of cultivation, market availability, or individual preferences. Consequently, the observed differences in CI cannot be attributed to these factors. The CI values are therefore interpreted as descriptive measures of reported utilization within the sampled population rather than as evidence of the underlying factors influencing plant selection or cultural importance.

### 4.3. Medicinal Knowledge

Medicinal use represented a relatively small component of the Verbenaceae uses documented in this study and was restricted to *Phyla nodiflora* and *Stachytarpheta jamaicensis*. This contrasts with the broader medicinal importance reported for members of Verbenaceae in the ethnobotanical literature from other regions [1,4]. Such a difference should not be interpreted as indicating that medicinal plant knowledge is generally limited in Buri Ram; rather, it reflects the family, taxa, informants, and community landscapes investigated in the present study.

The medicinal-use data also emphasize the importance of considering Fidelity Level together with the number of informants contributing to each reported use. For *P. nodiflora*, the highest FL was associated with treatment of chronic or purulent wounds and bruises, but medicinal use of this species was reported by a relatively small number of informants. The resulting percentage therefore represents agreement among those medicinal users rather than widespread consensus across the entire study population. For *S. jamaicensis*, medicinal reports involved a larger number of users and were distributed among several reported purposes. These differences are most appropriately interpreted as descriptive patterns in the medicinal knowledge documented during the survey. Accordingly, FL values are interpreted here descriptively and are not used to infer medicinal efficacy or population-wide consensus.

The medicinal applications recorded for these two species contribute to the documentation of local plant knowledge but do not establish pharmacological efficacy. Although medicinal uses of Verbenaceae, including Lantana camara and Stachytarpheta jamaicensis, have been reported in other ethnobotanical and traditional-medicine contexts, such records document reported or traditional use rather than, by themselves, demonstrating therapeutic efficacy [1,4,34,35].

Although *Verbena officinalis* L. is native to Thailand and has been reported from northern Thailand [7], it was not encountered in the community landscapes surveyed in Buri Ram Province. Its traditional medicinal uses have been documented in other regions [36] and are therefore discussed here only in the context of the published literature rather than as part of the present ethnobotanical findings. In addition, the potential toxicity of certain Verbenaceae warrants attention, particularly *Lantana camara*, which contains hepatotoxic pentacyclic triterpenoids known as lantadenes. Ingestion of this species has been associated with poisoning in grazing livestock [37]. Although a retrospective study of pediatric exposures reported predominantly no or minor clinical effects [38], accidental ingestion remains a relevant safety consideration in home gardens and residential environments where young children and domestic animals may have access to ornamental plants. As toxicity and poisoning incidents were not investigated in the present study, these considerations are included to raise awareness of potential hazards rather than to imply that adverse effects occurred in the surveyed communities.

### 4.4. Ethnolinguistic Significance of Vernacular Plant Names

The vernacular-name data demonstrate that local nomenclature does not necessarily correspond one-to-one with formal botanical or horticultural entities. This relationship is consistent with observations from folk-taxonomic studies in which vernacular classifications may organize plant diversity differently from scientific taxonomy [19]. The present study provides several examples of such relationships within Verbenaceae and illustrates the value of recording vernacular names together with taxonomically identified voucher material.

Morphological and sensory characteristics were prominent in informants’ explanations of the recorded names. References to inflorescence form, animals, familiar objects, flower color, fragrance, leaf appearance, and growth habit indicate that readily observable characteristics contribute to how the names are locally understood. Morphological and culturally meaningful attributes have similarly been reported as important components of Thai plant nomenclature [20,21]. Importantly, however, the explanations recorded in the present study represent informants’ interpretations of the names and do not by themselves establish their historical etymological origins.

Conceptually similar names were also recorded in Thai and in forms identified by informants as Thai-Khmer. The squirrel-tail concept associated with the inflorescences of *Aloysia virgata*, for example, occurred in both Thai and Thai-Khmer names. Comparable parallels were observed in the candle-related names associated with *Duranta erecta* ‘Dark Purple’ and the green-snake concept associated with *Stachytarpheta jamaicensis*. These observations demonstrate the occurrence of similar naming concepts in the recorded vocabulary but do not establish linguistic borrowing, direction of transmission, common historical origin, or association with ethnic groups. Such interpretations would require comparative linguistic and sociolinguistic evidence that was not collected in the present study [39,40].

The reported contexts in which names were encountered provide another distinction. Most names were described by informants as being conventionally used within their families and local communities, whereas Rachawadee, Bu-nga Sahree, and the *Verbena*-based names were reported as having been obtained from plant shops or vendors. The latter therefore represent horticulturally circulated terminology within the context of the present dataset. The data do not establish when or through what historical process these names entered local usage, and the plant trade should consequently not be assumed to represent their ultimate linguistic origin without additional historical and comparative linguistic evidence [19,41,42].

The relationship between vernacular naming and horticultural differentiation was also variable. The same vernacular name, *Phaga Grong*, was applied to *Lantana camara* and its recorded cultivars, whereas the flower-color variants of *Glandularia tenera* were differentiated by adding color terms to the horticultural name *Verbena*. *Lantana montevidensis*, in contrast, was distinguished through a name referring to its creeping habit. These examples demonstrate that locally expressed distinctions do not necessarily occur at the same level as botanical species, cultivar, or horticultural variant. A single vernacular name may encompass more than one horticulturally distinguishable entity, while conspicuous characteristics may generate modified names within another taxon. Similar differences between vernacular and scientific classification have been documented in folk-taxonomic systems elsewhere [19].

The etymological interpretations presented in this study should consequently be treated with appropriate caution. Where the historical or linguistic derivation of a name could not be independently verified, its explanation represents a locally reported interpretation rather than a definitive etymology. This is particularly important for names identified as Thai-Khmer language and for names that may have circulated through horticultural contexts. Formal reconstruction of their linguistic histories would require additional comparative, historical, and sociolinguistic evidence and specialist analysis [39,40,41,42]. The present approach instead preserves the meanings and explanations reported by informants while maintaining a distinction between contemporary local interpretation and demonstrated historical derivation.

### 4.5. Integration, Scope, and Implications

Taken together, the findings show that taxonomic occurrence, ecological occurrence, community recognition, utilization, cultural importance, and vernacular nomenclature represent related but distinct dimensions of Verbenaceae diversity in community landscapes. These dimensions were not consistently congruent. A horticultural entity could be botanically distinguishable without receiving a separate vernacular name, while another could be locally differentiated through flower color or growth habit. Likewise, widespread recognition did not necessarily correspond to a similarly high level of reported utilization. Integrating these components therefore provides information that would not be captured through occurrence records or ethnobotanical indices alone.

The interpretation of these findings must remain within the spatial and methodological scope of the study. Fieldwork targeted community and other human-influenced landscapes across all 23 districts and did not constitute a systematic survey of protected areas or natural forests. The recorded taxa therefore represent the Verbenaceae encountered within the targeted landscape types rather than the complete Verbenaceae flora of Buri Ram Province. Similarly, informants were purposively recruited in association with locations where the recorded taxa occurred. Recognition rates, use reports, and cultural indices consequently characterize the sampled community contexts and should not be extrapolated directly to the entire provincial population.

The study was designed to document community recognition, reported utilization, and vernacular nomenclature of Verbenaceae rather than to compare plant knowledge among demographic or ethnolinguistic groups. During fieldwork, overlapping linguistic practices were observed within the surveyed communities, although these observations were not part of a systematic sociolinguistic assessment. Accordingly, no demographic information, including age, sex, ethnicity, or educational background, was collected. This limitation prevents comparisons of plant knowledge among demographic groups and assessments of intergenerational knowledge transmission or potential knowledge loss. In particular, the identification of certain vernacular names as Thai-Khmer reflects their reported linguistic identity rather than the ethnic identity of the informants who used them. Nevertheless, the findings document how Verbenaceae, including introduced ornamental taxa, are recognized, named, and utilized within contemporary community landscapes, irrespective of their biogeographical origin. Future studies incorporating demographic and sociolinguistic information could further examine variation in plant knowledge and its transmission among community groups.

Occurrence records require a similar distinction. The absence of a taxon from the Flora of Thailand or from other sources examined in this study does not independently establish a first national or provincial record. Confirmation of such status, particularly for cultivated ornamentals, would require broader investigation of herbarium collections, the historical botanical and horticultural literature, and other occurrence records [27,28,29]. Likewise, the ecological categories assigned during fieldwork document the conditions under which taxa were encountered and do not establish temporal transitions among cultivated, spontaneous, and naturalized states.

Within these boundaries, the present study provides an integrated account of Verbenaceae in the community landscapes of Buri Ram. Voucher-based taxonomic documentation establishes which taxa were encountered, while occurrence information, recognition, reported utilization, cultural indices, and vernacular nomenclature reveal different aspects of their relationships with local communities. The results therefore demonstrate the value of considering taxonomic and horticultural diversity together with ethnobotanical and ethnolinguistic evidence when documenting plants in human-influenced landscapes, while maintaining clear distinctions between occurrence and new records, recognition and utilization, reported medicinal use and therapeutic efficacy, and locally interpreted name meanings and formal linguistic etymology.

### 4.6. Practical Significance of the Findings

The findings of this study provide a practical reference for botanical documentation, horticultural identification, community education, and future monitoring of Verbenaceae in human-influenced landscapes. The voucher-supported inventory, including introduced species, named cultivars, horticultural variants, and naturalized taxa, can assist local educators, horticultural practitioners, and community members in recognizing and distinguishing plants commonly encountered in residential and public spaces. The documentation of vernacular names, together with reported ornamental and medicinal uses, may also support locally relevant educational materials and the preservation of community-associated plant knowledge. From a public health perspective, the discussion of potential toxicity, particularly that of Lantana camara, highlights the importance of accurate plant identification and appropriate awareness of possible ingestion hazards. However, the reported medicinal uses should not be interpreted as evidence of clinical efficacy or safety. Furthermore, the voucher specimens and occurrence records provide baseline information for future assessments of cultivated and spontaneous populations, although the present data are insufficient for determining population trends or conservation status. These applications illustrate the value of integrating taxonomic, horticultural, and ethnobotanical evidence in community-based botanical research.

## 5. Conclusions

This study provides a voucher-based account of Verbenaceae occurring in community landscapes of Buri Ram Province, integrating taxonomic and horticultural diversity with ecological occurrence, community recognition, local utilization, and vernacular nomenclature. Sixteen taxa representing eight genera were documented, comprising species, an interspecific hybrid, named cultivars, and recognizable horticultural variants. The assemblage was predominantly composed of introduced and cultivated plants, while a small number of taxa also occurred under spontaneous or naturalized conditions. Community recognition and reported utilization varied considerably among taxa and did not necessarily correspond with one another. Ornamental use accounted for most use reports, whereas traditional medicinal knowledge was documented only for *Phyla nodiflora* and *Stachytarpheta jamaicensis*. The 22 vernacular names recorded further demonstrated that local naming may reflect morphological and sensory characteristics, metaphorical associations, horticultural terminology, and linguistic influences, while not necessarily corresponding one-to-one with formal botanical or horticultural entities.

Together, these findings broaden voucher-supported documentation of Verbenaceae in the community landscapes of Buri Ram and demonstrate the value of considering cultivated plants, horticultural entities, and locally recognized diversity alongside conventional floristic information. The study also highlights the importance of maintaining distinctions between ecological occurrence and confirmed distributional records, plant recognition and reported utilization, traditional medicinal knowledge and therapeutic efficacy, and locally reported interpretations of vernacular names and formal linguistic etymology. By integrating botanical and community-derived evidence within these interpretive boundaries, this study provides baseline information for future floristic, horticultural, ethnobotanical, and ethnolinguistic research on plant diversity in human-influenced landscapes. Future studies should incorporate demographic and sociolinguistic data, broader surveys of natural and human-influenced habitats, and repeated observations of cultivated and spontaneous populations to further investigate variation in plant knowledge, patterns of occurrence, and changes in the local distribution of Verbenaceae over time.

## Figures and Tables

**Table 1:** Taxonomic diversity, status, ecological occurrence, local utilization, and voucher specimens of Verbenaceae recorded in community landscapes of Buri Ram Province.

Taxon	Vernacular Name	Status for Thailand	Ecological Occurrences	Used Parts ^1^	Purposes of Use	Recorded in Flora of Thailand [7]	Voucher Specimens
*Aloysia virgata* (Ruiz & Pav.) Pers.	Rachawadee, Hang Kra Rok, Paga Gatui Gone Groke, Paga Gatui Ganghand	Introduced	Cultivated	Wp	Ornamental	No	VMSU38200011
*Citharexylum spinosum* L.	Bu-nga Sahree	Introduced	Cultivated	Wp	Ornamental	No	VMSU38200019
*Duranta erecta* ‘Dark Purple’	Tian Yot, Tian Hom, Dok Tian, Ten Gra Aob, Pa Ga Ten	Introduced	Cultivated	Wp	Ornamental	Yes	VMSU38200016
*Glandularia* × *hybrida* (Groenland & Rümpler) G.L.Nesom & Pruski	Verbena	Introduced	Cultivated	Wp	Ornamental	No	VMSU38200008
*Glandularia tenera* (Spreng.) Cabrera (Pink flowers)	Verbena Chomphoo	Introduced	Cultivated	Wp	Ornamental	No	VMSU38200018
*Glandularia tenera* (Spreng.) Cabrera (Purple flowers)	Verbena Muang	Introduced	Cultivated	Wp	Ornamental	No	VMSU38200017
*Glandularia tenera* (Spreng.) Cabrera (Red flowers)	Verbena Daeng	Introduced	Cultivated	Wp	Ornamental	No	VMSU38200009
*Lantana camara* L.	Phaga Grong	Introduced	Naturalized and spontaneous	Wp	Ornamental	Yes	VMSU38200006
*Lantana camara* ‘Mutabilis’	Phaga Grong	Introduced	Cultivated	Wp	Ornamental	No	VMSU38200013
*Lantana camara* ‘Nivea’	Phaga Grong	Introduced	Cultivated	Wp	Ornamental	No	VMSU38200010
*Lantana camara* ‘Sundancer’	Phaga Grong	Introduced	Cultivated	Wp	Ornamental	No	VMSU38200014
*Lantana montevidensis* (Spreng.) Briq.	Phaga Grong Luey	Introduced	Cultivated	Wp	Ornamental	No	VMSU38200012
*Petrea volubilis* L.	Phuang Kram	Introduced	Cultivated	Wp	Ornamental	No	VMSU38200005
*Petrea volubilis* ‘Albiflora’	Phuang Kram Khaow	Introduced	Cultivated	Wp	Ornamental	No	VMSU38200015
*Phyla nodiflora* (L.) Greene	Yah Gled Pla, Ton Gled Pla	Native	Spontaneous and cultivated	St	Traditional medicine	Yes	VMSU38200007
*Stachytarpheta jamaicensis* (L.) Vahl	Ton Ngu Kiaw, Puaa Keew	Introduced	Naturalized	Lv, St, Wp	Traditional medicine	Yes	VMSU38200004

^1^ Lv = Leaves, St = Stem; Wp = Whole plant.

**Table 2:** Community recognition, use reports, and Cultural Importance Index (CI) of Verbenaceae taxa recorded in community landscapes of Buri Ram, Eastern Thailand.

Taxon	*n*	RR (%)	Ornamental UR	Medicinal UR	CI-Ornamental	CI-Medicinal	Total CI
*Aloysia virgata*	109	23.7	52	0	0.113	0	0.113
*Citharexylum spinosum*	98	21.3	48	0	0.104	0	0.104
*Duranta erecta* ‘Dark Purple’	375	81.5	230	0	0.500	0	0.500
*Glandularia × hybrida*	71	15.4	71	0	0.154	0	0.154
*G. tenera* (pink)	66	14.3	66	0	0.143	0	0.143
*G. tenera* (purple)	78	17.0	78	0	0.170	0	0.170
*G. tenera* (red)	67	14.6	67	0	0.146	0	0.146
*Lantana camara*	408	88.7	128	0	0.278	0	0.278
*L. camara* ‘Mutabilis’	422	91.7	142	0	0.309	0	0.309
*L. camara* ‘Nivea’	337	73.3	31	0	0.067	0	0.067
*L. camara* ‘Sundancer’	389	84.6	23	0	0.050	0	0.050
*L. montevidensis*	333	72.4	7	0	0.015	0	0.015
*Petrea volubilis*	460	100.0	388	0	0.843	0	0.843
*P. volubilis* ‘Albiflora’	430	93.5	39	0	0.085	0	0.085
*Phyla nodiflora*	181	39.3	0	7	0	0.015	0.015
*Stachytarpheta jamaicensis*	313	68.0	0	28	0	0.061	0.061

Notes: *n* = number of informants recognizing each taxon; RR = Recognition Rate; UR = use report; CI = Cultural Importance Index; Total number of informants, *N* = 460. One use report represents one informant × one taxon × one use category.

**Table 3:** Medicinal uses and Fidelity Level (FL) of *Phyla nodiflora* and *Stachytarpheta jamaicensis* reported by informants in Buri Ram Province, Eastern Thailand.

Taxon	Part Used	Reported Medicinal Purpose	Preparation/Application	*Ip*	*Iu*	FL (%)
*Phyla nodiflora*	Stem	Fever	Dried stems boiled in water; decoction taken orally	2	7	28.6
	Stem	Coughing up blood	Dried stems boiled in water; decoction taken orally	1	7	14.3
	Stem	Hematuria/promotion of urination	Dried stems boiled in water; decoction taken orally	1	7	14.3
	Stem	Chronic/purulent wounds and bruises	Stems boiled; remaining plant material applied externally as a poultice	3	7	42.9
*Stachytarpheta jamaicensis*	Whole plant	Fever	Decoction/infusion; taken orally	2	28	7.1
	Whole plant	Urinary stones	Decoction/infusion; taken orally	3	28	10.7
	Whole plant	Joint pain	Infusion; taken orally	1	28	3.6
	Whole plant	Sore throat	Decoction/infusion; taken orally	1	28	3.6
	Whole plant	Inflamed wounds	Fresh plant material pounded and applied externally	4	28	14.3
	Whole plant	Ulcerative wounds	Fresh plant material pounded and applied externally	2	28	7.1
	Whole plant	Gastric disorders	Decoction/infusion; taken orally	3	28	10.7
	Whole plant	Intestinal worms	Decoction/infusion; taken orally	2	28	7.1
	Whole plant	Vomiting	Decoction/infusion; taken orally	3	28	10.7
	Stem	Diarrhea	Decoction/infusion; taken orally	2	28	7.1
	Stem	Dysentery	Decoction/infusion; taken orally	1	28	3.6
	Leaves	Muscular/body aches	Leaves rubbed or massaged externally	2	28	7.1
	Leaves	Abscesses/purulent lesions	Fresh material pounded and applied externally	2	28	7.1

Notes: *Ip* = number of informants reporting a particular medicinal purpose; *Iu* = total number of informants reporting any medicinal use of the taxon; FL = Fidelity Level.

**Table 4:** Vernacular names and ethnolinguistic characteristics of Verbenaceae recorded in Buri Ram Province, Eastern Thailand.

Taxon	Vernacular Name	IPA Transcription	Proper Meaning	Metonymical Meaning	Metaphorical Meaning	Notes
*Aloysia virgata*	Rachawadee	/raː tɕʰaː wa diː/	✓			“Rachawadee” was associated by informants with a royal connotation, approximately referring to something intended for or associated with royalty. The same vernacular name is also applied to *Buddleja paniculata* Wall., an ornamental species with superficially similar inflorescences. Its use for *A. virgata* was locally interpreted as a possible transfer of an established plant name based on perceived similarity.
*Aloysia virgata*	Hang Kra Rok	/hǎːŋ kra r$\hat{ɔ}$ːk/			✓	“Hang Kra Rok” consists of two Thai words: *Hang*, meaning “tail”, and *Kra Rok*, meaning “squirrel”. The name refers to the elongated inflorescences, which resemble a squirrel’s tail.
*Aloysia virgata*	Paga Gatui Gone Groke	/pa kaː ka tuj kon kroːk/			✓	Identified by informants as Thai-Khmer. According to their interpretation, *Paga* means “flower”, *Gatui* means “tail”, and *Gone Groke* means “squirrel”. The name therefore means “squirrel-tail flower”, referring to the elongated inflorescences.
*Aloysia virgata*	Paga Gatui Ganghand	/pa kaː ka tuj kaŋ hɛːn/			✓	Identified by informants as Thai-Khmer. According to their interpretation, *Paga* means “flower”, *Gatui* means “tail”, and *Ganghand* means “squirrel”. The name therefore means “squirrel-tail flower”, referring to the resemblance of the elongated inflorescences to a squirrel’s tail.
*Citharexylum spinosum*	Bu-nga Sahree	/bu ŋaː saː riː/			✓	According to informants, *Bu-nga* means “flower”, while *Sahree* refers to a sari. The long, pendulous white inflorescences are perceived as resembling the graceful hanging portion of a sari.
*Duranta erecta * ‘Dark Purple’	Tian Yot	/tʰian jòt/			✓	“Tian Yot” consists of two Thai words: *Tian*, meaning “candle”, and *Yot*, meaning “drop”. The rounded orange pendent fruits are perceived as resembling drops of orange candle wax.
*Duranta erecta * ‘Dark Purple’	Tian Hom	/tʰian h$\hat{ɔ}$ːm/		✓	✓	“Tian Hom” combines *Tian*, meaning “candle”, and *Hom*, meaning “fragrant”. The name retains the candle-related metaphor, while *Hom* refers directly to floral fragrance.
*Duranta erecta * ‘Dark Purple’	Dok Tian	/dɔːk̚˨˩ tʰia̯n˧/			✓	“Dok Tian” combines two Thai words: *Dok*, meaning “flower”, and *Tian*, meaning “candle”. The name retains the local candle-related association with the plant.
*Duranta erecta * ‘Dark Purple’	Ten Gra Aob	/ten kra ʔoːp/		✓	✓	Identified by informants as Thai-Khmer. According to their interpretation, *Ten* means “candle” and *Gra Aob* means “fragrant”. The name means “fragrant candle”, combining the candle-related metaphor with floral fragrance.
*Duranta erecta * ‘Dark Purple’	Pa Ga Ten	/pa kaː ten/			✓	Identified by informants as Thai-Khmer. According to their interpretation, *Pa Ga* means “flower” and *Ten* means “candle”, giving the meaning “candle flower”.
*Glandularia × hybrida*	Verbena	/wɤː biː naː/	✓			“Verbena” was reported as an established horticultural name used in the ornamental-plant trade. Informants associated its continued use with the former placement of these plants under *Verbena*.
*Glandularia tenera * (pink-flowered variant)	Verbena Chomphoo	/wɤː biː naː tɕʰom pʰuː/	✓	✓		“Verbena” is the established horticultural name, while the Thai word *Chomphoo* means “pink”. The modifier distinguishes the variant by its pink flowers.
*Glandularia tenera * (purple-flowered variant)	Verbena Muang	/wɤː biː naː mûaŋ/	✓	✓		“Verbena” is the established horticultural name, while *Muang* means “purple”. The modifier distinguishes the variant by its purple flowers.
*Glandularia tenera* (red-flowered variant)	Verbena Daeng	/wɤː biː naː dɛːŋ/	✓	✓		“Verbena” is the established horticultural name, while *Daeng* means “red”. The modifier distinguishes the variant by its red flowers.
*Lantana camara*	Phaga Grong	/pʰa kaː krɔːŋ/			✓	“Phaga Grong” combines *Phaga*, meaning “flower”, and *Grong*, referring to the stringing or ornamental arrangement of flowers. The name reflects the numerous small flowers densely arranged into rounded inflorescences.
*Lantana camara * ‘Mutabilis’	Phaga Grong	/pʰa kaː krɔːŋ/			✓	The same vernacular name as *L. camara* is used; no separate cultivar-specific name was recorded.
*Lantana camara* ‘Nivea’	Phaga Grong	/pʰa kaː krɔːŋ/			✓	The same vernacular name as *L. camara* is used; no separate cultivar-specific name was recorded.
*Lantana camara * ‘Sundancer’	Phaga Grong	/pʰa kaː krɔːŋ/			✓	The same vernacular name as *L. camara* is used; no separate cultivar-specific name was recorded.
*Lantana montevidensis*	Phaga Grong Luey	/pʰa kaː krɔːŋ l$\hat{ɯ}$aj/		✓	✓	The established name *Phaga Grong* is retained, while *Luey* refers to the creeping or trailing growth habit. The additional modifier distinguishes the species by habit.
*Petrea volubilis*	Phuang Kram	/pʰuaŋ kʰraːm/		✓		“Phuang Kram” combines *Phuang*, referring to a cluster or bunch, and *Kram*, meaning “indigo”. The name refers to the clustered inflorescences and their indigo coloration.
*Petrea volubilis* ‘Albiflora’	Phuang Kram Khaow	/pʰuaŋ kʰraːm kʰǎːw/		✓		The established name *Phuang Kram* is retained, while *Khaow* means “white”. The modifier distinguishes the cultivar by its white flowers.
*Phyla nodiflora*	Yah Gled Pla	/jâː klèt plaː/		✓	✓	“Yah Gled Pla” consists of three Thai words: *Yah*, referring to a weed or herb, *Gled*, meaning “scale”, and *Pla*, meaning “fish”. *Yah* reflects its recognition as a weedy herb, while *Gled Pla* metaphorically refers to leaves perceived as resembling fish scales.
*Phyla nodiflora*	Ton Gled Pla	/tôn klèt plaː/			✓	“Ton Gled Pla” consists of *Ton*, meaning “plant”, *Gled*, meaning “scale”, and *Pla*, meaning “fish”. The name refers to the perceived resemblance of the leaves to fish scales.
*Stachytarpheta jamaicensis*	Ton Ngu Kiaw	/tôn ŋuː kʰǐaw/		✓	✓	“Ton Ngu Kiaw” consists of three Thai words: *Ton*, meaning “plant”, *Ngu*, meaning “snake”, and *Kiaw*, meaning “green”. The elongated inflorescence is metaphorically associated with a snake, while *Kiaw* refers directly to its green color.
*Stachytarpheta jamaicensis*	Puaa Keew	/pua kʰiːw/			✓	Identified by informants as Thai-Khmer and interpreted by them as meaning “green snake”, *Puaa* meaning “snake”, and *Keew*, meaning “green”. The name refers to the elongated green inflorescence perceived as resembling a green snake. Individual lexical components were not independently established in this study.

Note: The table presents 22 vernacular names; repeated application of Phaga Grong to *Lantana camara* and its cultivars ‘Mutabilis’, ‘Nivea’, and ‘Sundancer’ was counted as a single vernacular name. IPA transcriptions represent the syllabic pronunciations provided by informants and transcribed during the interviews. Meanings and etymological explanations were recorded according to informants’ interpretations. Where the historical or linguistic origin of a term could not be independently verified, the explanation is treated as a local interpretation rather than a definitive etymological derivation. Linguistic attribution to Thai-Khmer refers to the reported linguistic origin of the name and does not imply the ethnicity of individual informants.

## Data Availability

The original contributions presented in this study are included in the article. Further inquiries can be directed to the corresponding authors.

## Supplementary Materials

The following supporting information can be downloaded at https://www.mdpi.com/article/10.3390/life16101745/s1, Supplementary File S1: Participant information, verbal consent script, and interview guide for the ethnobotanical survey of Verbenaceae in Buri Ram Province, Thailand.
