# Supplementary material for: Diversity, Local Utilization, and Ethnolinguistic Perspectives on Verbenaceae J.St.-Hil. in Community Landscapes of Buri Ram Province, Eastern Thailand
Source: Life (Basel). 2026 Oct 17;16(10):1745. doi: 10.3390/life16101745 (PMC13706457; doi:10.3390/life16101745)
Supplement: Supplementary file 1 [file life-16-01745-s001.zip › life-4633528-supplementary.pdf]

Verbal Consent Script

For ethnobotanical interviews concerning Verbenaceae in Buri Ram Province, Thailand

Study Project:	Diversity and Local Utilization of Verbenaceae J.St.-Hil. in Community Landscapes of Buri Ram, Eastern Thailand
Research team:	Surapon Saensouk, Piyaporn Saensouk, Thawatphong Boonma, Suriya Phimpha, Wei Shen, Pathomthat Srisuk and Auemporn Junsongduang
Document type:	Verbal consent script
Note:	This blank document is prepared for journal record purposes. It contains no personal or identifying information of any participant.

1. Purpose of the interview

The purpose of this interview is to document local knowledge concerning plants in the family Verbenaceae in Buri Ram Province, Thailand. The interview focuses on community recognition of the recorded plants, vernacular and alternative local or dialect names, local utilization, purposes of use, plant parts used, and, where applicable, methods of preparation or application. The interview focuses exclusively on plant-related knowledge and does not collect personally identifiable or demographic information, including age, sex, or ethnicity. The information collected will be used to examine relationships between Verbenaceae diversity, local plant utilization, and vernacular nomenclature in community landscapes.

2. Voluntary participation

Participation in this interview is voluntary. The participant may choose not to answer any question and may stop the interview at any time. Refusing to participate or stopping the interview will not cause any negative consequences.

3. Confidentiality and anonymity

No personal names, identification numbers, addresses, telephone numbers, photographs of participants, or other identifying information will be recorded. The information collected will be reported only in summarized scientific form.

4. Verbal consent procedure

Before starting the interview, the researcher explains the study objectives, the scope of the questions, voluntary participation, and confidentiality to the participant. The participant is then asked whether they agree to participate in the interview.

5. Verbal consent confirmation

Question asked before the interview:

Do you agree to participate in this interview and share your knowledge about Verbenaceae plants, including their local names and uses?

Response:

☐ Yes, verbal informed consent was obtained.

☐ No, the interview was not conducted.

6. Interview questions

No.	Topic	Interview question	Information recorded
**1**	Plant recognition	Do you recognize this plant?	Yes / No
**2**	Vernacular name	What do you call this plant?	Vernacular name(s) reported by the informant
**3**	Alternative/local name	Does this plant have any other local or dialect name(s)? If yes, what are they?	Alternative local or dialect name(s)
**4**	Name interpretation	If known, what does the name mean, or why is the plant called by this name?	Informant's interpretation of the meaning or origin of the name
**5**	Local utilization	Is this plant locally used?	Yes / No
**6**	Purpose of use	If yes, what is the plant used for?	Reported purpose or use category (e.g., ornamental or medicinal)
**7**	Plant part used	Which part(s) of the plant are used?	Whole plant, stem, leaves, or other reported part(s)
**8**	Preparation/application	How is the plant prepared or used for this purpose?	Method of preparation and/or application

Note: Living plants, where available, or photographs taken during the field surveys were presented to informants to facilitate recognition and ensure that the questions referred to the intended taxon. Questions 2–8 were asked only when the informant recognized the plant; questions concerning utilization (Questions 6–8) were asked only when a use was reported. Question 4 was asked where informants were able to provide an explanation and was treated as a locally reported interpretation rather than a definitive etymological derivation. The interviews focused exclusively on plant-related knowledge. No personally identifiable or demographic information, including age, sex, or ethnicity, was collected.

7. Researcher's record

Verbal informed consent was obtained before the interview. The participant was informed of the study objectives, voluntary participation, scope of questions, and confidentiality. No personal, identifying, or sensitive information was collected.

Verbal consent obtained	☐ Yes ☐ No
Date of interview	________________________
Researcher	________________________

8. Final note

This document is a blank participant information and verbal consent script prepared for journal record purposes. It does not contain personal or identifying information of any participant**.** It reflects the information explained to participants before conducting the ethnobotanical interviews.
